# Interactions of Surfactants with Biomimetic Membranes—2. Generation of Electric Potential with Non-Ionic Surfactants

**DOI:** 10.3390/membranes13030353

**Published:** 2023-03-18

**Authors:** Nikolai M. Kocherginsky, Brajendra K. Sharma

**Affiliations:** 1 NEXT-ChemX, Department of Chemistry, University of Illinois, Urbana, IL 61801, USA; 2 United States Department of Agriculture, Agricultural Research Service Eastern Regional Research Center, Wyndmoor, PA 19038, USA; brajendra.sharma@usda.gov

**Keywords:** surfactants, biomimetic membranes, transmembrane transport, electric potential, oscillations

## Abstract

It is known that noncharged surfactants lead to electric effects that interact with biomimetic membranes made of nitrocellulose filters, which are impregnated with fatty acid esters. At a surfactant concentration as low as 64 micrometers in one of the solutions, they lead to the transient formation of transmembrane electric potential. Maximum changes of this potential are proportional to the log of noncharged surfactant concentrations when it changes by three orders of magnitude. We explain this new and nontrivial effect in terms of an earlier suggested physicochemical mechanics approach and noncharged surfactants transient changes induced by membrane permeability for inorganic ions. It could be used to imitate the interactions of non-ionic drugs with biological membranes. The effect may also be used in determining the concentration of these surfactants and other non-ionic chemicals of concern, such as pharmaceuticals and personal care products.

## 1. Introduction

Different biomimetic membranes [1,2,3] can be used for ex situ prebiological drug screening and to predict their penetration and interactions with biological membranes [4].

Previously, we suggested using biomimetic membranes made of nitrocellulose filters impregnated with free fatty acids, their esters or vegetable oils. These membranes are easy to make and are much more stable than lipid monolayers and bilayers. Simultaneously they have polysaccharide polymer chains, analogs of lipids, and spontaneously formed cation-selective aqueous nanochannels at the interface of hydrophilic polymer and oil. Ionic selectivity is determined by impurities of carboxylic groups fixed on a relatively hydrophilic polymer matrix, which used to be a major component of wood. These aqueous nanochannels with ion-exchanging groups explain why these membranes have many specific (per unit of thickness) properties mimicking those of biological membranes, which are impossible with many other model membranes. Examples are transmembrane electric resistance and capacitance, frequency-dependent impedance, cation/anion and even cation/cation selectivity, permeability and selectivity for important respiratory gases CO_2_/O_2,_ the permeability of water, transport of small hydrophilic molecules in aqueous channels and larger hydrophobic molecules in oils, the possibility of facilitated and active ion transport, possibility to selectively regulate permeability through the pores filled with hydrophobic fatty acids and thin aqueous channels, oscillations of electric properties near the melting point of fatty acids, and even correlations of induced by several psychotropic drugs membrane lysis and nonspecific hepatotoxicity in hospitals-all these effects are observed without proteins in our membranes. This is a fundamental difference between traditional bilayer lipid membranes and liposomes, where proteins are added to obtain functionality. The membranes are ~ times thicker than biological membranes, but their many specific (after recalculation to the same thickness) fundamental physicochemical properties are similar to those of biomembranes. Therefore, our membranes may be called biomimetic membranes and are discussed in more detail in a recent review [3], where many references to original papers may be found.

The application of surfactants in the global market has increased steadily in pharmaceutical, detergent, cosmetic, paint, food science, nanotechnology, petroleum recovery, bioremediation, chemical transformation, and drug delivery [5]. Earlier work by Rosen et al. [6] supported the hypothesis that interfacial properties, such as adsorption and penetration through cell membranes, determine the biological toxicity of anionic and nonionic surfactants. In our previous article, we described the interactions of ionic surfactants with oil-impregnated nitrocellulose filters [7]. When cationic cetyl ammonium bromide (CTAB) was added into one of the solutions separated by the membrane, it led to several interesting effects that were dependent on concentration. After the addition of the surfactant at relatively low concentrations, transient kinetics of transmembrane electric potential was observed. The potential of the donor solution decreased but then reached a steady state, which is explained by the redistribution of organic cations between the solution and membrane. In the case of anionic dodecyl sulfate, the sign of potential changes was the opposite, which may be explained by penetration into the membrane of organic ions with different charges. When the concentration of the surfactants in the solution was increased, after several hours of incubation, it was possible to observe spontaneous transmembrane electric oscillations of voltage and current. At even higher concentrations of surfactants, oil was washed out from the membrane pores, and the membrane lost its barrier properties. As a result, the transmembrane difference of electric potentials disappeared, and membrane conductivity increased by two orders of magnitude.

Here, we present an extension of that approach to nonionic surfactants. Non-charged surfactants are attracting a lot of attention in the industry [8,9,10,11,12,13]. The properties and applications of non-ionic amphoteric surfactants are discussed in a recent review [14]. When we used noncharged surfactants at concentrations above the critical micelle concentration, washing out of the impregnating liquid (laundry effect) was also observed. Surprisingly, at lower concentrations, noncharged surfactants lead to the transient formation of transmembrane electric potential and then even to oscillations of electric properties. Below, we describe the nontrivial effect of transmembrane voltage formation induced by noncharged surfactants, which has not been previously observed.

## 2. Materials and Methods

The methods used were described previously [7]. Millipore-mixed cellulose filters (0.45 μ and 0.05 μ pore size) were impregnated with liquid isopropyl myristate and placed vertically in a Teflon chamber, separating the chamber into two cylindrical halves. After less than a minute, the pores of the filter were filled with the oil, which can be easily confirmed by the weight difference before and after impregnation. The filter thickness was measured by micrometer and was nearly 110 microns.

Ag/AgCl electrodes were inserted into each of them, separated by the membrane solutions. The measuring (right) half-chamber, where the surfactant was added later, was filled with 12 mL of 5 mM potassium dihydrogen phosphate (KH_2_PO_4_). The reference (left) half-chamber was filled with 5 mM KH_2_PO_4_ + 0.5 M KCl. pH of both solutions was adjusted to 4.6. The temperature was maintained at 25 °C with the water bath. Stirring was carried out only in the measuring chamber at 50 rpm using a magnetic stirrer. Initially, we waited for 30 min. After the potential of a measuring Ag/AgCl electrode stabilized, we added the surfactant into this right solution and started data acquisition.

**Figure 1:**
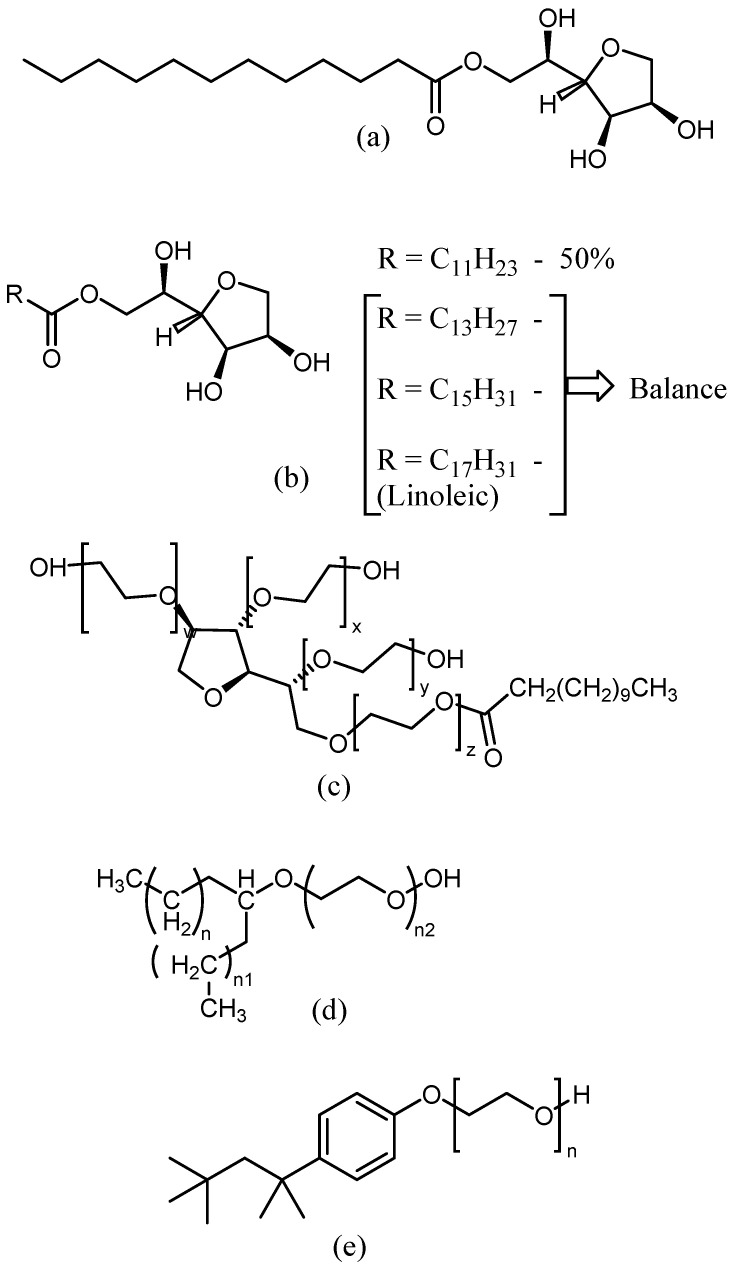
Structures of nonionic surfactants (**a**) sorbitan monolaurate Span-20, (**b**) other sorbitans, (**c**) polyethylene glycol sorbitan monolaurate, Tween 20, (**d**) Tergitol, and (**e**) Triton X-100.

Commercial nonionic surfactants, sorbitan monolaurate Span-20 (Figure 1a) and other Spans (Figure 1b), polyethylene glycol sorbitan monolaurate Tween 20 (Figure 1c), were from Sigma-Aldrich (St. Louis, MO, USA) and made by Dow (Midland, MI, USA). Secondary alcohol ethoxylate Tergitol 15-S-7 (Figure 1d) and Triton X-100 (Figure 1e) were used without special treatment. Initially, these surfactants were dissolved in deionized water to make an aqueous 5 *w/v*% solution.

## 3. Results

In the presence of different K^+^ concentrations, due to the K^+^ penetration from the donor (left) 0.5 M KCl solution to the 5 mM KH_2_PO_4_ acceptor solution, the electric potential in the acceptor solution was near +120 mV versus the donor solution. This value is expected based on the Nernst equation. The membrane is cation/anion-selective, and the chloride practically did not penetrate because of repulsion by negatively charged carboxylic groups in aqueous nanochannels. When a similar experiment was carried out in symmetric conditions (only buffer in both chambers), ion concentration was the same on both sides, and the initial transmembrane potential was zero.

**Figure 2:**
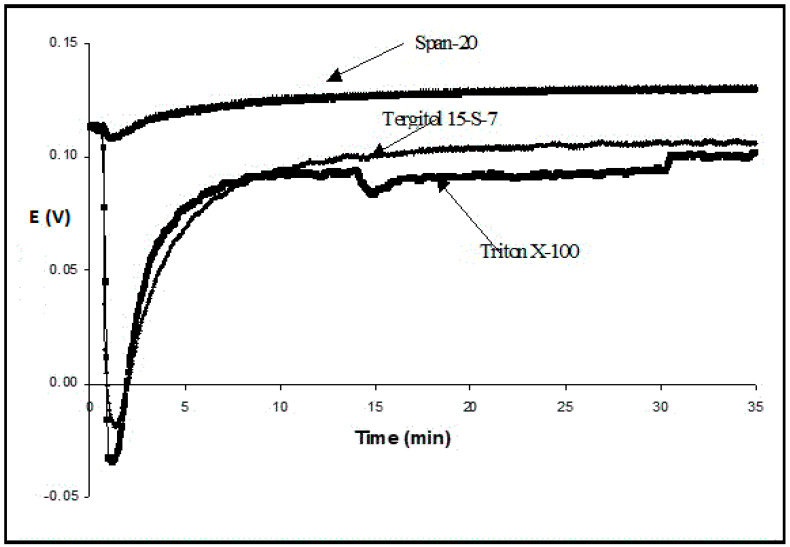
Transmembrane potential vs. time after the addition of 0.1 *w/v*% of different surfactants to the measuring (K^+^ acceptor) chamber containing 5 mM buffer. The reference (K^+^ donor) chamber contains 5 mM buffer + 0.5 M KCl.

As soon as 0.1 *w/v*% Tergitol 15-S-7 was added into the acceptor (measuring) solution with a lower concentration of K^+^, there was a sharp drop in this potential, which reached –20 mV after less than 2 min. Immediately afterward, it started increasing until it reached almost an initial value. This transient process took approximately twenty minutes. Similar effects were observed with Triton X-100, Tween 20, and Tween 80 (Figure 2).

The effects were much less with Span-20, which belongs to the class of anhydrosorbitol esters and does not have polyethoxy chains, whereas Tergitol and Triton have them. The hydrophilic–lipophilic balance (HLB) value of Span-20 is 8.6—the smallest among other surfactants used, but it is still capable of forming oil in a water emulsion [15]. Porcine bile extract (a very mild natural surfactant) also led to a minimum of the potential, which then returned to the initial value, but all stages were milder and slower. For example, it took more than 30 min to reach the minimum potential for a 1.5% solution.

**Figure 3:**
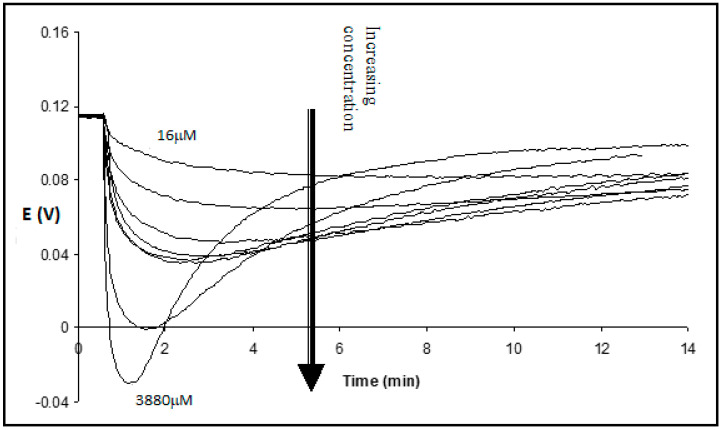
Transmembrane potential vs. time after the addition of Tergitol 15-S-7 in concentrations 16, 32, 64, 97, 130, 162, 800, and 3880 μM to the measuring (acceptor) chamber, containing the 5 mM buffer. The reference (donor) chamber contains 5 mM buffer + 0.5 M KCl.

**Figure 4:**
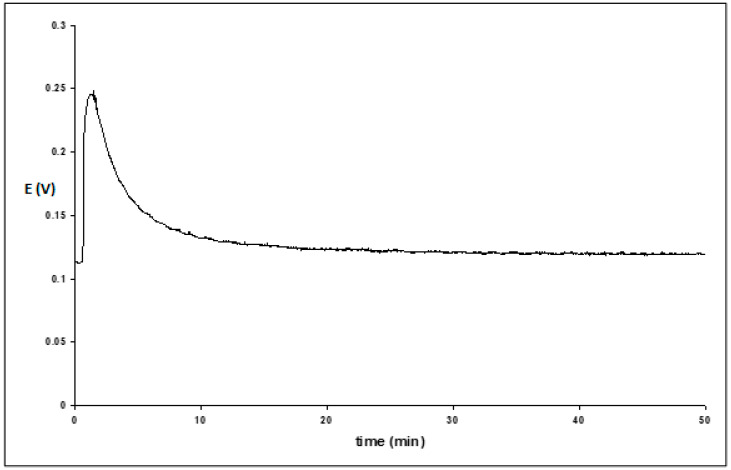
Transmembrane potential as a function of time when Tergitol 15-S-7 was added to the reference chamber. The measuring chamber contains a 5 mM buffer, while the reference chamber contains 5 mM buffer + 0.5 M KCl, pH = 4.6.

After the addition of Tergitol, the drop in transmembrane potential decreased with the decrease in surfactant concentration (Figure 3). It took more time to reach the minimum and to return. When Tergitol was added to the opposite solution with the reference electrode, the transient kinetics changed its sign, but finally, as expected, the potential practically returned to the initial value (Figure 4).

**Figure 5:**
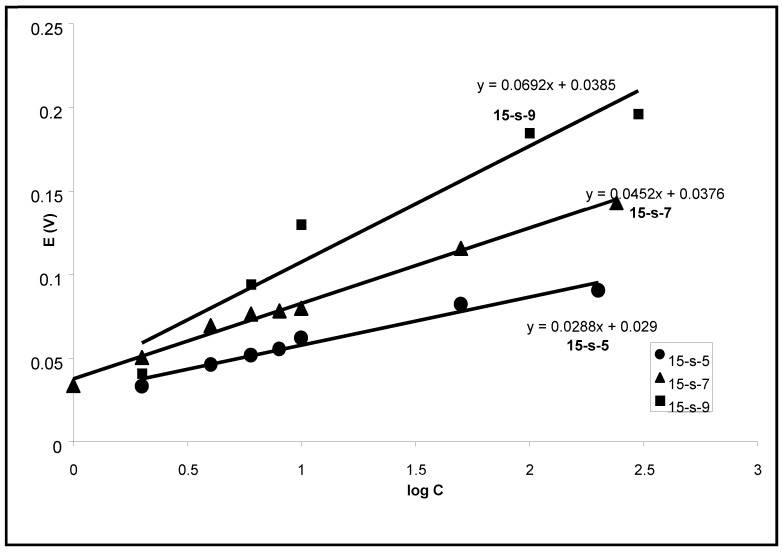
Net changes of the minimum value of the transmembrane potential as a function of log C (µm) for different Tergitols: ● 15-S-5, ▲ 15-S-7 and ■ 15-S-9.

**Figure 6:**
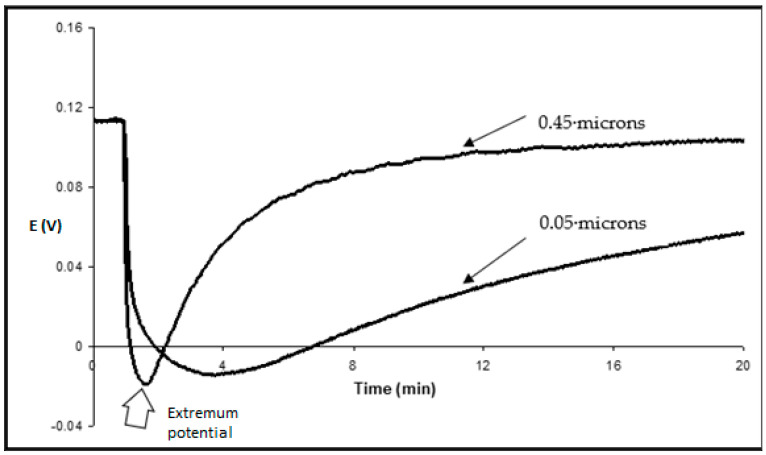
Transmembrane potential as a function of time for impregnated nitrocellulose filters with pore size 0.05 μm and 0.45 μm.

Changes in the transmembrane potential from the initial value to the minimum were proportional to log C when the surfactant concentration changed by three orders of magnitude. The slope of this dependence increased with the length of a chain in surfactant, and it was 69 mV per 10-fold increase of Tergitol 15-S9 concentration (Figure 5). The preliminary addition of 0.5 M KCl into the measuring solution, where the surfactant was later added, led to a simple downward shift of the kinetics (potential changes are larger) but did not change the slope of concentration dependence. In its turn, when the pore size of the filter was decreased from 0.45 μ to 0.05 μ, the dependence of transmembrane potential changes on log C was also practically the same, but the kinetics were slower (Figure 6).

**Figure 7:**
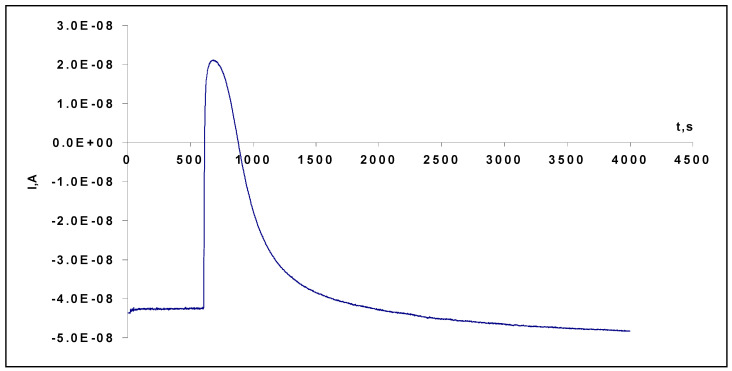
Time dependence of current through the membrane in response to 3.5 mM Tween 20. 0.45 μm nitrocellulose filter impregnated with isopropyl myristate separated by a 5 mM buffer+ 0.5 M KCl and a 5 mM buffer.

Transient effects were also observed when we measured the transmembrane electric current. The results are presented for Tween 20 (Figure 7). Initially, the current was negative (positive charges move from the acceptor solution with more positive potential), but after the addition of the surfactant, it changed from −4 × 10^−8^ A to + 2 × 10^−8^ A at maximum, then finally returned to the initial value. Measured separately, membrane resistance was near 2 MΩ. Based on the maximum current increase by 6 × 10^−8^ A, this corresponds to experimental changes in the potential near 120 mV. In comparison, the electric resistance of impregnated hydrophobic Teflon membranes, which do not have aqueous channels, is 100–1000 times more.

## 4. Discussion

There are two major possible reasons why noncharged surfactants induce transient changes in electric potential. The first is the direct interactions of K^+^ with -OCH_2_CH_2_- or other groups in the surfactant molecules. Another is based on the surfactant interactions with the membrane, influencing ion transport. The second mechanism is confirmed by the fact that not only porcine bile extract but even the addition of propanol leads to the transient formation of minimum potential. The initial phase, in this case, was several times faster than for the nonionic surfactants.

The distribution of an ion *i* between ideal and homogeneous aqueous and membrane phases is determined by its electrochemical potential. At equilibrium, it should be the same in aqueous (*w*) and membrane (*m*) phases:

(1) $$\mu_{0i}(w)+RT\ln c_{i}(w)+zF\psi (w)=\mu_{0i}(m)+RT\ln c_{i}(m)+zF\psi (m)$$

Thus, ion distribution is determined by both the difference in electric potentials ψ(m)−ψ(w) and the difference in the standard chemical potentials $\mu_{0i}(m)-\mu_{0i}(w)$. If the noncharged surfactant is distributed between the aqueous solution and the membrane, it locally modifies its properties, so that $\mu_{0i}(m)$ becomes dependent on the local concentration of the surfactant and its distance from the membrane surface *x*. This is the major reason why a noncharged surfactant influences ion distribution and, as a result, creates a transmembrane difference in electric potential. Initially, after the addition of the surfactant into one of the solutions, the system was not in equilibrium. The ions are redistributed with time together with the redistribution of the noncharged surfactant, changing the transmembrane potential.

The physicochemical model explaining these observations may be based on the equation describing the flux *J* (mol/cm^2^ s) of an ion *i* as a function of its local concentration $c_{i}(x)$ and the gradient of $\partial \mu_{gi}/\partial x$ [16]:

(2) $$J_{i}(x)=-U_{i}c_{i}\frac{\partial \mu_{gi}}{\partial x}=-U_{i}c_{i}\frac{\partial (\mu_{0i}+RT\ln\gamma_{i}+RT\ln c_{i}+zF\psi +etc.)}{\partial x}$$

Here, we added the activity coefficient $\gamma_{i}$ and the possibility of other driving factors, which may be, for example, a transmembrane pressure difference. As a result, the local flux is proportional to the product of local concentration and total acting molar force $-\partial \mu_{gi}/\partial x$ with the units Newton/mol. For transport, we need total molar force and concentration. The proportionality coefficient $U_{i}$ was suggested by A. Einstein to describe diffusion and is called mobility (see later edition of his papers) [17]. Here $\mu_{gi}$ is what we call a general physicochemical potential, i.e., the total molar Gibbs energy of an ion *i* in the presence of all possible driving factors, such as concentration, nonideality, electric field, pressure, magnetic field, surface tension, etc. In ideal conditions, without additional driving factors, $\mu_{gi}$ it is reduced to traditional electrochemical potential $\mu_{0i}+RT\ln c_{i}+zF\psi$. At a constant temperature, it includes three driving factors, i.e., the gradients of $\mu_{0i}$, $RT\ln c_{i}$, and electric potential ψ. In a homogeneous phase, a standard chemical potential $\mu_{0i}$ and activity coefficient $\gamma_{i}$ are constant, but they change and become dependent on *x* when the added surfactant enters the membrane from one side, so the membrane becomes asymmetric.

In the steady state, $J_{i}=const\neq f(x,time)$. When the temperature does not depend on *x*, Equation (1) may be integrated from x=0 to x=l giving (we drop the subscript *i*.)

(3) $$J_{x}=-\frac{\exp\frac{\mu_{g}(l)}{RT}-\exp\frac{\mu_{g}(0)}{RT}}{\int_{0}^{l}\frac{\exp(\frac{\mu_{f}}{RT})}{RTU(x)}dx}$$

$\mu_{f}$ is a force potential, which includes all driving factors but not the term with concentration. Previously, it was shown that Equations (1) and (2) together lead to practically all major transport and equilibrium equations, including Fick’s law, Ohm’s law, etc. [16]. For example, it is easy to see that in a simple case when $\mu_{gi}=\mu_{0}{}_{i}+RT\ln c_{i}+F\psi$ and $J_{x}=0$ (equilibrium) Equation (2) is reduced to the Nernst equation. After the substitution of expressions for $\mu_{gi}$ and $\mu_{f}$ it is also easy to see that RTU(x) is the local diffusion coefficient, which is known as the Planck–Einstein relation.

To describe ion transport influenced by simultaneously changing concentration gradients of nonionic surfactants, one equation is not enough—we must add one more Equation (1), but without ψ, which describes transmembrane transport of nonionic surfactant. Furthermore, we also assume that the local standard potential $\mu_{0}{}_{i}(x)$ of an ion in the membrane decreases proportionally to the log of the local concentration of surfactant $c{\left(x\right)}_{\mathrm{surfactant}}$. In other words, the surfactant locally modifies the membrane, which becomes more hydrophilic and has a higher affinity to the metal ions. The solution to this system of equations is rather complicated, but it is possible to suggest a qualitative prediction. When the surfactant at a low concentration enters the membrane, it is initially located near the membrane surface. Induced by this surfactant, a decrease of local $\mu_{0i}(x)$ for K^+^ serves as an additional driving factor, leading to K^+^ redistribution into the membrane. One of the reasons for this redistribution is that surfactants increase the accessibility of negatively charged carboxylic groups present as a fixed impurity on a nitrocellulose polymer matrix. Changes in Log$c{\left(x\right)}_{\mathrm{surfactant}}$ will lead to linear changes of $\mu_{g}$ K^+^ in the membrane and its distribution. Thus, a modification of the membrane from one (acceptor) side and the entrance of K^+^ ions from this side led to changes in the Donnan surface potential. Measured with an Ag/AgCl electrode, the electric potential in the acceptor solution becomes more negative and proportional to the log$c{\left(x\right)}_{\mathrm{surfactant}}$, which is what was observed in the experiment (Figure 5). Thus, the induced membrane asymmetry explains the initial sharp transitory decrease of transmembrane potential.

If the surfactant concentration is not too high, i.e., less than the one leading to oscillations, phase transitions, and washing out of oil from pores, the membrane retains its barrier properties. With time, the surfactant diffuses through the oil-filled pores, and its concentration gradient in the membrane decreases. This leads to the decrease of a driving force for K^+^ transport inside the membrane $-d\mu_{0i}(x)/dx$ and then to symmetric changes of the Donnan potential on the donor side. The changes in K^+^ concentrations in both solutions are small, and the final transmembrane potential returns practically to the initial value described by the Nernst equation for a symmetric membrane (Figure 3).

**Figure 8:**
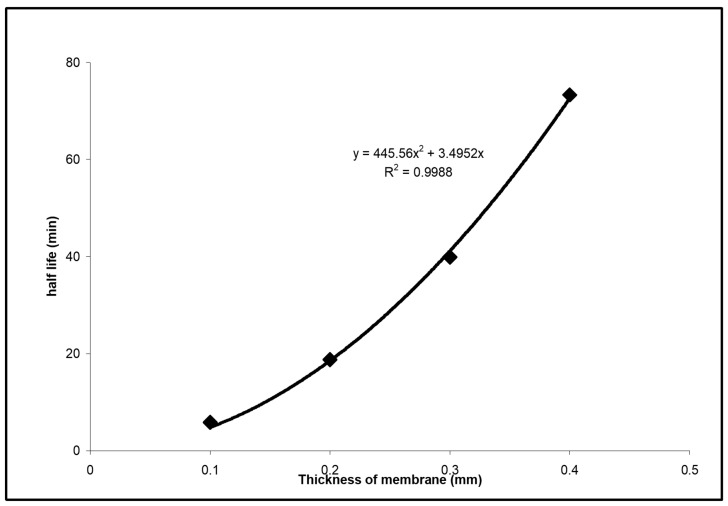
The half-life time of potential relaxation kinetics vs. the thickness of the membrane with 0.05-micrometer pores after the addition of 0.1 wt% Tergitol 15-S-7.

The kinetics of electric potential relaxation practically to the initial value should be determined by diffusion of noncharged surfactant. In the simplest case of a homogeneous membrane and fast transition through the interface, its characteristic time should be described by

(4) $$\tau =\frac{l^{2}}{6D}$$

where D is the diffusion coefficient of surfactant in the membrane [17,18]. In separate experiments, the membrane thickness *l* was varied from approximately 100 μ to 400 μ, with the stack of one, two, three and four impregnated filters, and relaxation kinetics was characterized by time *τ*_1/2_ necessary to decrease the difference of final and minimum potential by half. For one filter with 0.05-micrometer pores and 0.1 wt% of Tergitol, this half-life time was nearly 4 min, but it was proportional to *l^2^* and increased to more than 65 min for four filters (Figure 8). Calculated based on Equation (3) diffusion coefficient was 7 × 10^−8^ cm^2^/s. It seems possible that nonionic surfactants are penetrating via aqueous nanochannels, which explains the relatively high diffusion coefficients. The concentration gradient of the noncharged surfactant will lead to the gradient of the standard chemical potential of ions $-d\mu_{0i}(x)/dx$ and to the gradient of surface tension in the nanochannel, which will serve as additional driving factors for ion transport. This reminds pressure gradients in a capillary in traditional electrokinetic effects like streaming current and steaming potential, though, of course, the hydrodynamic description is different. Here we should also mention that facilitated by fatty acids, H^+^ transport is not described by simple Equation (3) because it is determined by two diffusion coefficients, one diffusion coefficient of H^+^ in the aqueous channel with ion exchange and another for the fatty acid, which serves as an H^+^ carrier [19]. Moreover, if the membrane is initially asymmetric, the characteristic time decreases because, in this case, it is determined not only by diffusion but also by a drift due to the preexisting gradient $-d\mu_{0i}(x)/dx$ [20].

In membrane science, it is assumed that membrane properties are not modified by permeating molecules. This approximation is not valid in the case of biomimetic membranes, and it is natural to assume that similar effects are possible in much thinner biological membranes, explaining why ion transport may be regulated and even coupled with the transport of neutral drug molecules.

Finally, a few words regarding possible practical applications. In a previous paper [7], we mentioned that our experimental system and surfactant-induced processes were similar to washing in a laundry machine and can be used to screen new surfactants and detergents. In addition, the fact that the extremum changes in the transmembrane electric potential are proportional to the log of uncharged surfactant concentration when it changes by three orders of magnitude may be useful in the analytical chemistry of these surfactants, which is usually done by titration.

## 5. Conclusions

We demonstrate that adding noncharged molecules of surfactants from one side of the biomimetic membranes influences ion transport and leads to transitory changes in the transmembrane voltage and current. We explained these effects based on the previously suggested physicochemical mechanics approach and introduced membrane asymmetry, which changes while the neutral surfactant penetrates from one solution to another through the membrane. They are also important for asymmetric biological membranes [21] and their interactions with noncharged molecules, including non-ionic chemicals like pharmaceuticals and personal care products. The described effects may be used as a simple electrochemical approach in the analytical chemistry of noncharged molecules in water. Currently, this is usually done by time- and labor-consuming titration [22,23].

## Data Availability

Not applicable.

## References

[1] Nielsen C.H. (2009). Biomimetic membranes for sensor and separation applications. Anal. Bioanal. Chem..

[2] Shen Y.-X., Saboe P.O., Sines I.T., Erbakan M., Kumar M. (2014). Biomimetic membranes: A review. J. Membr. Sci..

[3] Kocherginsky N.M. (2021). Biomimetic membranes without proteins but with aqueous nanochannels and facilitated transport. Minireview. Membr. Membr. Technol..

[4] Avdeef A. (2003). Absorption and Drug Development.

[5] Sar P., Ghosh A., Scarso A., Saha B. (2019). Surfactant for better tomorrow: Applied aspect of surfactant aggregates from laboratory to industry. Res. Chem. Intermed..

[6] Rosen M.J., Fei L., Zhu Y.P., Morrall S.W. (1999). The relationship of the environmental effect of surfactants to their interfacial properties. J. Surfact. Deterg..

[7] Kocherginsky N., Sharma B.K. (2021). Interactions of Surfactants with Biomimetic Membranes. 1. Ionic Surfactants. J. Surfact. Deterg..

[8] Schick M.J. (1987). Nonionic Surfactants: Physical Chemistry.

[9] Van Os N.M., Haak J.R., Rupert L.A.M. (1993). Physico-Chemical Properties of Selected Anionic, Cationic, and Nonionic Surfactants.

[10] Van Os N.M. (1998). Nonionic Surfactants: Organic Chemistry.

[11] Nace V.M. (1996). Nonionic Surfactants: Polyoxyalkylene Block Copolymers.

[12] Balzer D., Lüders H. (2000). Nonionic Surfactants: Alkyl Polyglucosides.

[13] Hayes D.G., Solaiman D.K.Y., Ashby R.D. (2019). Biobased Surfactants: Synthesis, Properties, and Applications.

[14] Sarkar R., Pal A., Rakshit A., Saha B. (2021). Properties and applications of amphoteric surfactant: A concise review. J. Surfact. Deterg..

[15] Rosen M.J., Kunjappu J.T. (2012). Surfactants and Interfacial Phenomena.

[16] Kocherginsky N.M., Gruebele M. (2016). Mechanical approach to chemical transport. Proc. Natl. Acad. Sci. USA.

[17] Einstein A. (1956). Investigations on the Theory of the Brownian Movement.

[18] Cussler E.L. (1997). Diffusion: Mass Transfer in Fluid Systems.

[19] Kocherginsky N.M., Osak I.S. (1986). Dependence of the time lag necessary to establish a steady state rate of transport in a liquid membrane on the concentration of a “carrier”. Russian J. Phys. Chem..

[20] Kocherginsky N.M., Zhang Y.K. (2003). Role of standard chemical potential in transport through anisotropic media and asymmetrical membranes. J. Phys. Chem. B.

[21] Kotyk A., Janaĉek K. (1977). Membrane Transport. An Interdisciplinary Approach.

[22] Cross J. (1986). Nonionic Surfactants: Chemical Analysis.

[23] Schmitt T. (2001). Analysis of Surfactants.

